# Comparison Between Linear and Non-parametric Regression Models for Genome-Enabled Prediction in Wheat

**DOI:** 10.1534/g3.112.003665

**Published:** 2012-12-01

**Authors:** Paulino Pérez-Rodríguez, Daniel Gianola, Juan Manuel González-Camacho, José Crossa, Yann Manès, Susanne Dreisigacker

**Affiliations:** * Colegio de Postgraduados, Montecillo, Texcoco 56230, México; † Departments of Animal Sciences, Dairy Science, and Biostatistics and Medical Informatics, University of Wisconsin-Madison, Madison, Wisconsin 53706; ‡ Biometrics and Statistics Unit and Global Wheat Program, International Maize and Wheat Improvement Center (CIMMYT), 06600 Mexico, D.F., México

**Keywords:** GenPred, Shared data resources

## Abstract

In genome-enabled prediction, parametric, semi-parametric, and non-parametric regression models have been used. This study assessed the predictive ability of linear and non-linear models using dense molecular markers. The linear models were linear on marker effects and included the Bayesian LASSO, Bayesian ridge regression, Bayes A, and Bayes B. The non-linear models (this refers to non-linearity on markers) were reproducing kernel Hilbert space (RKHS) regression, Bayesian regularized neural networks (BRNN), and radial basis function neural networks (RBFNN). These statistical models were compared using 306 elite wheat lines from CIMMYT genotyped with 1717 diversity array technology (DArT) markers and two traits, days to heading (DTH) and grain yield (GY), measured in each of 12 environments. It was found that the three non-linear models had better overall prediction accuracy than the linear regression specification. Results showed a consistent superiority of RKHS and RBFNN over the Bayesian LASSO, Bayesian ridge regression, Bayes A, and Bayes B models.

Genome-enabled prediction of complex traits based on marker data are becoming important in plant and animal breeding, personalized medicine, and evolutionary biology (Meuwissen *et al.* 2001; Bernardo and Yu 2007; de los Campos *et al.* 2009, 2010; Crossa *et al.* 2010, 2011; Ober *et al.* 2012). In the standard, infinitesimal, pedigree-based model of quantitative genetics, the family structure of a population is reflected in some expected resemblance between relatives. The latter is measured as an expected covariance matrix among individuals and is used to predict genetic values (*e.g.* Crossa *et al.* 2006; Burgueño *et al.* 2007, 2011). Whereas pedigree-based models do not account for Mendelian segregation and the expected covariance matrix is constructed using assumptions that do not hold (*e.g.* absence of selection and mutation and random mating), the marker-based models allow tracing Mendelian segregation at several positions of the genome and observing realized (as opposed to expected) covariances. This enhances the potential for improving the accuracy of estimates of genetic values, thus increasing the genetic progress attainable when these predictions are used for selection purposes in lieu of pedigree-based predictions. Recently, de los Campos *et al.* (2009, 2010) and Crossa *et al.* (2010, 2011) used Bayesian estimates from genomic parametric and semi-parametric regressions, and they found that models that incorporate pedigree and markers simultaneously had better prediction accuracy for several traits in wheat and maize than models based only on pedigree or only on markers.

The standard linear genetic model represents the phenotypic response of the *i^th^* individual ($y_{i}$) as the sum of a genetic value, $g_{i}$, and of a model residual, $\varepsilon_{i}$, such that the linear model for *n* individuals (i=1,…,n) is represented as $y_{i}=g_{i}+\varepsilon_{i}$. However, building predictive models for complex traits using a large number of molecular markers (*p*) with a set of lines comprising individuals (*n*) with *p ≫n* is challenging because individual marker effects are not likelihood-identified. In this case, marker effects can be estimated via penalized parametric or semi-parametric methods or their Bayesian counterparts, rather than via ordinary least squares. This reduces the mean-squared error of estimates; it also increases prediction accuracy of out-of-sample cases and prevents over-fitting (de los Campos *et al.* 2010). In addition to the well-known Bayes A and B linear regression models originally proposed by Meuwissen *et al.* (2001) for incorporating marker effects into $g_{i}$, there are several penalized parametric regression methods for estimating marker effects, such as ridge regression, the least absolute shrinkage and selection operator (LASSO), and the elastic net (Hastie *et al.* 2009). The Bayesian counterparts of these models have proved to be useful because appropriate priors can be assigned to the regularization parameter(s), and uncertainty in the estimations and predictions can be measured directly by applying the Bayesian paradigm.

Regression methods assume a linear relationship between phenotype and genotype, and they typically account for additive allelic effects only; however, evidence of epistatic effects on plant traits is vast and well documented (*e.g.* Holland 2001, 2008). In wheat, for instance, detailed analyses have revealed a complex circuitry of epistatic interactions in the regulation of heading time involving different vernalization genes, day-length sensitivity genes, and earliness *per se* genes, as well as the environment (Laurie *et al.* 1995; Cockram *et al.* 2007). Epistatic effects have also been found to be an important component of the genetic basis of plant height and bread-making quality traits (Zhang *et al.* 2008; Conti *et al.* 2011). It is becoming common to study gene × gene interactions by using a paradigm of networks that includes aggregating gene × gene interaction that exists even in the absence of main effects (McKinney and Pajewski 2012). Interactions between alleles at two or more loci could theoretically be represented in a linear model via use of appropriate contrasts. However, this does not scale when the number of markers (*p*) is large, as the number of 2-locus, 3-locus, *etc*., interactions is mind boggling.

An alternative approach to the standard parametric modeling of complex interactions is provided by non-linear, semi-parametric methods, such as kernel-based models (*e.g.* Gianola *et al.* 2006; Gianola and van Kaam 2008) or artificial neural networks (NN) (Okut *et al.* 2011; Gianola *et al.* 2011), under the assumption that such procedures can capture signals from high-order interactions. The potential of these methods, however, depends on the kernel chosen and on the neural network architecture. In a recent study, Heslot *et al.* (2012) compared the predictive accuracy of several genome-enabled prediction models, including reproducing kernel Hilbert space (RKHS) and NN, using barley and wheat data; the authors found that the non-linear models gave a modest but consistent predictive superiority (as measured by correlations between predictions and realizations) over the linear models. In particular, the RKHS model had a better predictive ability than that obtained using the parametric regressions.

The use of RKHS for predicting complex traits was first proposed by Gianola *et al.* (2006) and Gianola and van Kaam (2008). de los Campos *et al.* (2010) further developed the theoretical basis of RHKS with “kernel averaging” (simultaneous use of various kernels in the model) and showed its good prediction accuracy. Other empirical studies in plants have corroborated the increase in prediction accuracy of kernel methods (*e.g.* Crossa *et al.* 2010, 2011; de los Campos *et al.* 2010; Heslot *et al.* 2012). Recently, Long *et al.* (2010), using chicken data, and González-Camacho *et al.* (2012), using maize data, showed that NN methods provided prediction accuracy comparable to that obtained using the RKHS method. In NN, the bases functions (adaptive “covariates”) are inferred from the data, which gives the NN great potential and flexibility for capturing complex interactions between input variables (Hastie *et al.* 2009). In particular, Bayesian regularized neural networks (BRNN) and radial basis function neural networks (RBFNN) have features that make them attractive for use in genomic selection (GS).

In this study, we examined the predictive ability of various linear and non-linear models, including the Bayes A and B linear regression models of Meuwissen *et al.* (2001); the Bayesian LASSO, as in Park and Casella (2008) and de los Campos *et al.* (2009); RKHS, using the “kernel averaging” strategy proposed by de los Campos *et al.* (2010); the RBFNN, proposed and used by González-Camacho *et al.* (2012); and the BRNN, as described by Neal (1996) and used in the context of GS by Gianola *et al.* (2011). The predictive ability of these models was compared using a cross-validation scheme applied to a wheat data set from CIMMYT’s Global Wheat Program.

## Materials and Methods

### Experimental data

The data set included 306 elite wheat lines, 263 lines that are candidates for the 29^th^ Semi-Arid Wheat Screening Nursery (SAWSN), and 43 lines from the 18^th^ Semi-Arid Wheat Yield Trial (SAWYT) from CIMMYT’s Global Wheat Program. These lines were genotyped with 1717 diversity array technology (DArT) markers generated by Triticarte Pty. Ltd. (Canberra, Australia; http://www.triticarte.com.au). Two traits were analyzed: grain yield (GY) and days to heading (DTH) (see Supporting Information, File S1).

The traits were measured in a total of 12 different environments (1–12) (Table 1): GY in environments 1–7 and DTH in environments 1–5 and 8–12 (10 in all). Different agronomic practices were used. Yield trials were planted in 2009 and 2010 using prepared beds and flat plots under controlled drought or irrigated conditions. Yield data from experiments in 2010 were replicated, whereas data from trials in 2009 were adjusted means from an alpha lattice incomplete block design with adjustment for spatial variability in the direction of rows and columns using the autoregressive model fitted in both directions.

**Table 1:** Twelve environments representing combinations of diverse agronomic management (drought or full irrigation, sowing in standard, bed, or flat systems), sites in Mexico, and years for two traits, grain yield (GY) and days to heading (DTH), with their broad-sense heritability (*h*^2^) measured in 2010

Environment Code	Agronomic Management	Site in Mexico	Year	Trait Measured	*h*^2^ (GY)	*h*^2^ (DTH)
1	Drought-bed	Cd. Obregon	2009	GY, DTH	—	—
2	Drought-bed	Cd. Obregon	2010	GY, DTH	0.833	0.991
3	Drought-flat	Cd. Obregon	2010	GY, DTH	0.465	0.984
4	Full irrigation-bed	Cd. Obregon	2009	GY, DTH	—	—
5	Full irrigation-bed	Cd. Obregon	2010	GY, DTH	0.832	0.086
6	Heat-bed	Cd. Obregon	2010	GY	0.876	—
7	Full irrigation-flat melga	Cd. Obregon	2010	GY	0.876	—
8	Standard	Toluca	2009	DTH	—	—
9	Standard	El Batan	2009	DTH	—	—
10	Small observation plot	Cd. Obregon	2009	DTH	—	—
11	Small observation plot	Cd. Obregon	2010	DTH	—	0.950
12	Standard	Agua Fria	2010	DTH	—	0.990

Data used to train the models for GY and DTH in 2009 were the best linear unbiased estimator (BLUE) after spatial analysis, whereas the BLUE data for 2010 were obtained after performing analyses in each of the 12 environments and combined. The experimental designs in each location consisted of alpha lattice incomplete block designs of different sizes, with two replicates each.

Broad-sense heritability at individual environments was calculated as $h^{2}=\sigma_{g}^{2}/(\sigma_{g}^{2}+\frac{\sigma_{e}^{2}}{nreps})$, where $\sigma_{g}^{2}$ and $\sigma_{e}^{2}$ are the genotype and error variance components, respectively, and *nreps* is the number of replicates. For the combined analyses across environments, broad-sense heritability was calculated as $h^{2}=\sigma_{g}^{2}/(\sigma_{g}^{2}+\frac{\sigma_{ge}^{2}}{nenv}+\frac{\sigma_{e}^{2}}{nenv\times nreps)})$, where the term $\sigma_{ge}^{2}$ is the genotype × environment interaction variance component, and *nenv* is the number of environments included in the analysis.

### Statistical models

One method for incorporating markers is to define $g_{i}$ as a parametric linear regression on marker covariates $x_{ij}$ with form $g_{i}=\sum_{j=1}^{p}x_{ij}\beta_{j}$, such that $y_{i}=\sum_{j=1}^{p}x_{ij}\beta_{j}+\varepsilon_{i}$ ( *j* = 1,2,…,*p* markers); here, $\beta_{j}$ is the partial regression of $y_{i}$ on the *j^th^* marker covariate (Meuwissen *et al.* 2001). Extending the model to allow for an intercept

$$y_{i}=\mu +\sum_{j=1}^{p}x_{ij}\beta_{j}+\varepsilon_{i}$$ (1)

We adopted Gaussian assumptions for model residuals; specifically, the joint distribution of model residuals in Equation 1 was assumed normal with mean zero and variance $\sigma_{e}^{2}$. The likelihood function is

$$p\left(y|\mu ,g,\sigma_{e}^{2}\right)=\prod_{i=1}^{n}N\left(y_{i}|\mu +\sum_{j=1}^{p}x_{ij}\beta_{j},\sigma_{e}^{2}\right)$$ (2)

where $N\left(y_{i}|\mu +\sum_{j=1}^{p}x_{ij}\beta_{j},\sigma_{e}^{2}\right)$ is a normal density for random variable $y_{i}$ centered at $\mu +\sum_{j=1}^{p}x_{ij}\beta_{j}$ and with variance $\sigma_{e}^{2}$. Depending on how priors on the marker effects are assigned, different Bayesian linear regression models result.

### Linear models: Bayesian ridge regression, Bayesian LASSO, Bayes A, and Bayes B

A standard penalized regression method is ridge regression (Hoerl and Kennard 1970); its Bayesian counterpart, Bayesian ridge regression (BRR), uses a prior density of marker effects, $p\left(\beta_{j}|\omega \right)$, that is, Gaussian, centered at zero and with variance common to all the markers, that is, $p\left(\beta_{j}|\sigma_{\beta }^{2}\right)=N\left(\beta_{j}|0,\sigma_{\beta }^{2}\right)$, where $\sigma_{\beta }^{2}$ is a prior-variance of marker effects. Marker effects are assumed independent and identically distributed *a priori*. We assigned scaled inverse chi distributions $\chi^{-2}(df_{.},s_{.})$ to the variance parameters $\sigma_{e}^{2}$ and $\sigma_{\beta }^{2}$. The prior degrees of freedom parameters were set to $df_{.}=4$ and $s_{.}=1$. It can be shown that the posterior mean of marker effects is the best linear unbiased predictor (BLUP) of marker effects, so Bayesian ridge regression is often referred to as RR-BLUP (de los Campos *et al.* 2012).

The Bayesian LASSO, Bayes A, and Bayes B relax the assumption of common prior variance to all marker effects. The relationship among these three models is as follows: Bayes B can be considered as the most general of the three, in the sense that Bayes A and Bayesian ridge regression can be viewed as special cases of Bayes B. This is because Bayes A is obtained from Bayes B by setting π=0 (the proportion of markers with null effects), and Bayesian ridge regression is obtained from Bayes B by setting π=0 and assuming that all the markers have the same variance.

Bayes B uses a mixture distribution with a mass at zero, such that the (conditional) prior distribution of marker effects is given by

$$\beta_{j}|\sigma_{j}^{2},\pi =\{\begin{array}{ll} 0 & \text{with probability }\pi \\ N\text{(0,}\sigma_{j}^{2}\text{)} & \text{with probability 1-}\pi \end{array}$$ (3)

The prior assigned to $\sigma_{j}^{2},\;j=1,\ldots .,p$ is the same for all markers, *i.e.* a scaled inverted chi squared distribution $\chi^{-2}(df_{\beta },s_{\beta })$, where $df_{\beta }$ are the degrees of freedom and $s_{\beta }$ is a scaling parameter. Bayes B becomes Bayes A by setting *π* = 0.

In the case of Bayes B, we took π=0.95, $df_{\beta }=4$, and $s_{\beta }={\tilde{\sigma }}_{a}^{2}(df_{\beta }-2)/df_{\beta }$ with ${\tilde{\sigma }}_{a}^{2}={\tilde{\sigma }}_{S}^{2}/\left[(1-\pi )\sum_{j=1}^{p}2q_{j}(1-q_{j})\right]$, where $q_{j}$ is the allele frequency for marker *j* and ${\tilde{\sigma }}_{S}^{2}$ is the additive genetic variance explained by markers [see Habier *et al.* (2011) and Resende *et al.* (2012) for more details]. In the case of $\sigma_{e}^{2}$, we assigned a flat prior as in Wang *et al.* (1994).

The Bayesian LASSO assigns a double exponential (DE) distribution to all marker effects (conditionally on a regularization parameter λ), centered at zero and with marker-specific variance, that is, $p\left(\beta_{j}|\lambda ,\sigma_{e}\right)=DE\left(\beta_{j}|0,\frac{\lambda }{\sigma_{e}^{2}}\right)$. The DE distribution does not conjugate with the Gaussian likelihood, but it can be represented as a mixture of scaled normal densities, which allows easy implementation of the model (Park and Casella 2008; de los Campos *et al.* 2009). The priors used were exactly the same as those used in González-Camacho *et al.* (2012).

The models used in this study, the Bayesian ridge regression, Bayesian LASSO (BL), Bayes A, and Bayes B, are explained in detail in several articles; for example, Bayes A and Bayes B are described in Meuwissen *et al.* (2001), Habier *et al.* (2011), and Resende *et al.* (2012), and an account of BL is given in de los Campos *et al.* (2009, 2012), Crossa *et al.* (2010, 2011), Perez *et al.* (2010), and González-Camacho *et al.* (2012).

### Non-linear models: RBFNN, BRNN, and RKHS

In this section, we describe the basic structure of the non-linear single hidden layer feed-forward neural network (SLNN) with two of its variants, the radial basis function neural network and the Bayesian regularized neural network. We also give a brief explanation of RKHS with the averaging kernel method at the end of this section.

#### Single hidden layer feed-forward neural network:

**Figure 1:**
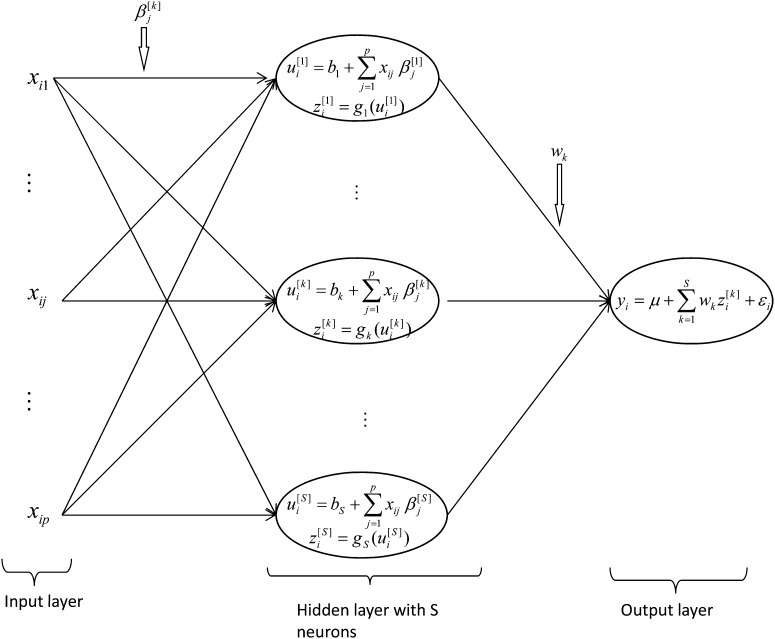
Structure of a single-layer feed-forward neural network (SLNN) adapted from González-Camacho *et al.* (2012). In the hidden layer, input variables $x_{i}$ = $\left(x_{i1},\ldots ,x_{ip}\right)$ (*j = 1,…,p* markers) are combined for each neuron (*k=*1,…,*S* neurons) using a linear function, $u_{i}^{\left[k\right]}=b_{k}+\sum_{j=1}^{p}x_{ij}\;\beta_{j}^{\left[k\right]}$, and subsequently transformed using a non-linear activation function, yielding a set of inferred scores, $z_{i}^{\left[k\right]}=g_{k}\left(u_{i}^{\left[k\right]}\right)$. These scores are used in the output layer as basis functions to regress the response using the linear activation function on the data-derived predictors $y_{i}=\mu +\sum_{k=1}^{S}w_{k}z_{i}^{\left[k\right]}+\varepsilon_{i}$.

In a single-layer feed-forward (SLNN), the non-linear activation functions in the hidden layer enable a NN to have universal approximation ability, giving it great potential and flexibility in terms of capturing complex patterns. The structure of the SLNN is depicted in Figure 1, which illustrates the structure of the method for a phenotypic continuous response. This NN can be thought of as a two-step regression (*e.g.* Hastie *et al.* 2009). In the first step, in the non-linear hidden layer, *S* data-derived basis functions (*k* = 1, 2,…, *S* neurons), $\left\{z_{i}^{\left[k\right]}\right\}$, are inferred, and in the second step, in the linear output layer, the response is regressed on the basis functions (inferred in the hidden layer). The inner product between the input vector and the weight vector ($\beta^{\left[k\right]}$) of each neuron of the hidden layer, plus a bias (intercept $b_{k}$), is performed, that is, $u_{i}^{\left[k\right]}=b_{k}+\sum_{j=1}^{p}x_{ij}\;\beta_{j}^{\left[k\right]},$ (*j* = 1,…,*p* markers); this is then transformed using a non-linear activation function $g_{k}(u_{i}^{\left[k\right]})$. One obtains $z_{i}^{\left[k\right]}=g_{k}\left(b_{k}+\sum_{j=1}^{p}x_{ij}\;\beta_{j}^{\left[k\right]}\right)$, where $b_{k}$ is an intercept and (*β*_1_^[^*^1^*^]^, …, *β_p_*^[^*^1^*^]^ ;…, *β*_1_^[^*^S^*^]^, …, *β_p_*^[^*^S^*^]^)′ is a vector of regression coefficients or “weights” of each neuron *k* in the hidden layer. The $g_{k}(.)$ is the activation function, which maps the inputs into the real line in the closed interval [−1,1]; for example, $g_{k}(x)=\frac{\exp(2x)-1}{\exp(2x)+1}$ is known as the tangent hyperbolic function. Finally, in the linear output layer, phenotypes are regressed on the data-derived features, $\left\{z_{i}^{\left[k\right]}\right\}$, according to

$$y_{i}=\mu +\sum_{k=1}^{S}w_{k}z_{i}^{\left[k\right]}+\varepsilon_{i}=\mu +\sum_{k=1}^{S}w_{k}g_{k}\left(b_{k}+\sum_{j=1}^{p}x_{ij}\beta_{j}^{\left[k\right]}\right)+\varepsilon_{i}.$$ (4)

#### Radial basis function neural network:

**Figure 2:**
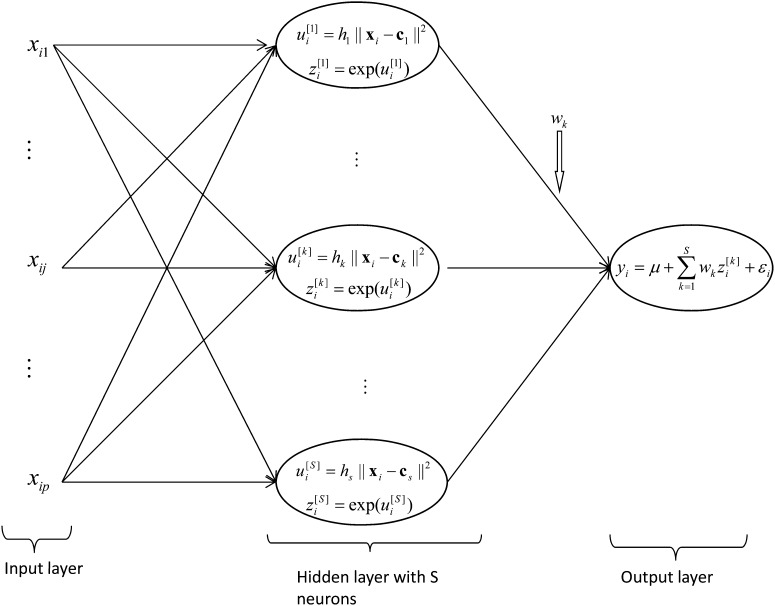
Structure of a radial basis function neural network adapted from González- Camacho *et al.* (2012). In the hidden layer, information from input variables $\left(x_{i1},\ldots ,x_{ip}\right)$ (*j = 1,…,p* markers) is first summarized by means of the Euclidean distance between each of the input vectors $\left\{x_{i}\right\}$ with respect to *S* (data-inferred) (*k=*1,…,*S* neurons) centers $\left\{c_{k}\right\}$, that is, $u_{i}^{\left[k\right]}=h_{k}||x_{i}-c_{k}|{|}^{2}$. These distances are then transformed using the Gaussian function $z_{i}^{\left[k\right]}=\text{exp}(-u_{i}^{\left[k\right]})$. These scores are used in the output layer as basis functions for the linear regression $y_{i}=\mu +\sum_{k=1}^{S}w_{k}z_{i}^{\left[k\right]}+\varepsilon_{i}$.

The RBFNN was first proposed by Broomhead and Lowe (1988) and Poggio and Girosi (1990). Figure 2 shows the architecture of a single hidden layer RBFNN with *S* non-linear neurons. Each non-linear neuron in the hidden layer has a Gaussian radial basis function (RBF) defined as $z_{i}^{\left[k\right]}=\text{exp}[-h_{k}{\left\|x_{i}-c_{k}\right\|}^{2}]$, where $\;\left\|x_{i}-c_{k}\right\|$ is the Euclidean norm between the input vector $x_{i}$ and the center vector $c_{k}$ and $h_{k}$ is the bandwidth of the Gaussian RBF. Subsequently, in the linear output layer, phenotypes are regressed on the data-derived features, $\left\{z_{i}^{\left[k\right]}\right\}$, according to $y_{i}=\mu +\sum_{k=1}^{S}w_{k}z_{i}^{\left[k\right]}+\varepsilon_{i}$, where $\varepsilon_{i}$ is a model residual.

##### Estimating the parameters of the RBFNN:

The vector of weights $\omega =\left\{w_{1},\ldots ,w_{S}\right\}$ of the linear output layer is obtained using the ordinary least-squares fit that minimizes the mean squared differences between the ${\hat{y}}_{i}\;$ (from RBFNN) and the observed responses $y_{i}\;$in the training set, provided that the Gaussian RBFs for centers $c_{k}$ and $h_{k}$ of the hidden layer are defined. The centers are selected using an orthogonalization least-squares learning algorithm, as described by Chen *et al.* (1991) and implemented in Matlab 2010b. The centers are added iteratively such that each new selected center is orthogonal to the others. The selected centers maximize the decrease in the mean-squared error of the RBFNN, and the algorithm stops when the number of centers (neurons) added to the RBFNN attains a desired precision (*goal* error) or when the number of centers is equal to the number of input vectors, that is, when *S = n*. The bandwidth $h_{k}$ of the Gaussian RBF is defined in terms of a design parameter of the net *spread*, that is, $h_{k}={\left(\frac{0.8326}{spread}\right)}^{2}$ for each Gaussian RBF of the hidden layer. To select the best RBFNN, a grid for training the net was generated, containing different values of *spread* and different precision values (*goal* error). The initial value of the *spread* was the median of the Euclidean distances between each pair of input vectors ($X_{i}$), and an initial value of 0.02 for the *goal* error was considered. The parameter *spread* allows adjusting the form of the Gaussian RBF such that it is sufficiently large to respond to overlapping regions of the input space but not so big that it might induce the Gaussian RBF to have a similar response.

#### Bayesian regularized neural networks:

The difference between SLNN and BRNN is in the function to be minimized (see the penalized function below); therefore, the basic structure of a BRNN can be represented in Figure 1 as well. The SLNN described above is flexible enough to approximate any non-linear function; this great flexibility allows NN to capture complex interactions among predictor variables (Hastie *et al.* 2009). However, this flexibility also leads to two important issues: (1) as the number of neurons increases, the number of parameters to be estimated also increases; and (2) as the number of parameters rises, the risk of over-fitting also increases. It is common practice to use penalized methods via Bayesian methods to prevent or palliate over-fitting.

MacKay (1992, 1994) developed a framework for obtaining estimates of all the parameters in a feed-forward single neural network by using an empirical Bayes approach. Let θ=(*w*_1_,…,*w_S_*; *b*_1_,…,*b_S_* ; *β*_1_^[1]^, …, *β_p_*^[1]^ ;…, *β*_1_^[^*^S^*^]^, …, *β_p_*^[^*^S^*^]^, *µ*)′ be the vector containing all the weights, biases, and connection strengths. The author showed that the estimation problem can be solved in two steps, followed by iteration:

(1) Obtain the conditional posterior modes of the elements in θ assuming that the variance components $\sigma_{e}^{2}$ and $\sigma_{\theta }^{2}$ are known and that the prior distribution for the all the elements in θ is given by $p(\theta |\sigma_{\theta }^{2})=MN(0,\sigma_{\theta }^{2}I)$. It is important to note that this approach assigns the same prior to all elements of θ, even though this may not always be the best thing to do. The density of the conditional (given the variance parameters) posterior distribution of the elements of θ, according to Bayes’ theorem, is given by
  $$p(\theta |y,\sigma_{e}^{2},\sigma_{\theta }^{2})=\frac{p(y|\theta ,\sigma_{e}^{2})p(\theta |\sigma_{\theta }^{2})}{p(y|\sigma_{e}^{2},\sigma_{\theta }^{2})}$$ (5)

The conditional modes can be obtained by maximizing Equation 5 over θ. However, the problem is equivalent to minimizing the following penalized sum of squares [see Gianola *et al.* (2011) for more details]

$$F(\theta )=\beta \sum_{i=1}^{n}e_{i}^{2}+\alpha \sum_{j=1}^{m}\theta_{j}^{2}$$

where $\beta =1/(2\sigma_{e}^{2})$, $\alpha =1/(2\sigma_{\theta }^{2})$, $e_{i}$ is the difference between observed and predicted phenotypes for the fitted model, and $\theta_{j}$ (j=1,…,m) is the *j^th^* element of vector θ.

(2) Update $\sigma_{e}^{2}$ and$\sigma_{\theta }^{2}$. The updating formulas are obtained by maximizing an approximation to the marginal likelihood of the data $p(y|\sigma_{e}^{2},\sigma_{\theta }^{2})$ (the “evidence”) given by the denominator of Equation 5.
(3) Iterate between (1) and (2) until convergence.

The original algorithm developed by MacKay was further improved by Foresee and Hagan (1997) and adopted by Gianola *et al.* (2011) in the context of genome and pedigree-enabled prediction. The algorithm is equivalent to estimation via maximum penalized likelihood estimation when “weight decay” is used, but it has the advantage of providing a way of setting the extent of “weight decay” through the variance component$\sigma_{\theta }^{2}$. Neal (1996) pointed out that the procedure of MacKay (1992, 1994) can be further generalized. For example, there is no need to approximate probabilities via Gaussian assumptions; furthermore, it is possible to estimate the entire posterior distributions of all the elements in θ, not only their (conditional) posterior modes. Next, we briefly review Neal’s approach to solving the problem; a comprehensive revision can be found in Lampinen and Vehtari (2001).

##### Prior distributions:

##### a) Variance component of the residuals:

Neal (1996) used a conjugate inverse Gamma distribution as a prior for the variance associated with the residual, $\varepsilon_{i}$, given in Equation 4, that is, $\sigma_{e}^{2}∼\text{Inv-Gamma}(s_{e},df_{e})$, where $s_{e}$ and $df_{e}$ are the scale and degrees of freedom parameters, respectively. These parameters can be set to the default values given by Neal (1996), $s_{e}$=0.05, $df_{e}$=0.5. These values were also used by Lampinen and Vehtari (2001).

##### b) Connection strengths, weights, and biases:

Neal (1996) suggested dividing the network parameters in θ into groups and then using hierarchical models for each group of parameters; for example, connection strengths (*β*_1_^[1]^, …, *β_p_*^[^*^1^*^]^ ;…; *β_1_*^[^*^S^*^]^, …, *β_p_*^[^*^S^*^]^), biases (*b*_1_,…,*b_S_*) of the hidden layer, and output weights (*w_1_*,…,*w_S_*), and general mean or bias (*µ*) of the linear output layer. Suppose that *u*_1_,…,*u_k_* are parameters of a given group; then assume

$$p(u_{1},\ldots ,u_{k}|\sigma_{u}^{2})={\left(2\pi \right)}^{-k/2}\sigma_{u}^{k}\exp\left\{-\frac{1}{2\sigma_{u}^{2}}\sum_{k=1}^{S}u_{k}^{2}\right\}$$

And, at the last stage of the model, assign the prior $\sigma_{u}^{2}∼\text{Inv-Gamma}(s_{u},df_{u})$. The scale parameter of the distribution associated with the group of parameters containing the connection strengths (*β*_1_*^[^*^1]^, …, *β_p_*^[1]^ ;…; *β*_1_^[^*^S^*^]^, …, *β_p_*^[^*^S^*^]^) changes according to the number of inputs, in this case, $s_{u}={\left(0.05/p^{1/df_{u}}\right)}^{2}$ with $df_{u}=0.5$ and *p* is the number of markers in the data set.

By using Markov chain Monte Carlo (MCMC) techniques through an algorithm called hybrid Monte Carlo, Neal (1996) developed a software termed flexible Bayesian modeling (FBM) capable of obtaining samples from the posterior distributions of all unknowns in a neural network (as in Figure 1).

#### Reproducing kernel Hilbert spaces regression:

RKHS models have been suggested as an alternative to multiple linear regression for capturing complex interaction patterns that may be difficult to account for in a linear model framework (Gianola *et al.* 2006). In RKHS model, the regression function takes the form

$$f\left(x_{i}\right)=\mu +\sum_{i^{\prime }=1}^{n}\alpha_{i^{\prime }}K\left(x_{i},x_{i^{\prime }}\right)$$ (6)

where $x_{i}={\left(x_{i1},\ldots ,x_{ip}\right)}^{\prime }$ and $x_{i^{\prime }}={\left(x_{i^{\prime }1},\ldots ,x_{i^{\prime }p}\right)}^{\prime }$ are input vectors of marker genotypes in individuals *i* and *i′*; $\alpha_{i^{\prime }}$ are regression coefficients; and $K\left(x_{i},x_{i^{\prime }}\right)=\exp\left(-h{\left\|x_{i}-x_{i^{\prime }}\right\|}^{2}\right)$ is the reproducing kernel defined (here) with a Gaussian RBF, where h is a bandwidth parameter and $\left\|x_{i}-x_{i^{\prime }}\right\|$ is the Euclidean norm between each pair of input vectors. The strategy termed “kernel averaging” for selecting optimal values of h within a set of candidate values was implemented using the Bayesian approach described in de los Campos *et al.* (2010). Similarities and connections between the RKHS and the RBFNN are given in González-Camacho *et al.* (2012).

### Assessment of the models’ predictive ability

The predictive ability of the models given above was compared using Pearson’s correlation and predictive mean-squared error (PMSE) using predicted and realized values. A total of 50 random partitions were generated for each of the data sets, and each partition randomly assigned 90% of the lines to the training set and the remaining 10% to the validation set. The partition scheme used was similar to that in Gianola *et al.* (2011) and González-Camacho *et al.* (2012).

All scripts were run in a Linux work station; for Bayesian ridge regression and Bayesian LASSO, we used the R package BLR (de los Campos and Perez 2010), whereas for RKHS, we used the R implementation described in de los Campos *et al.* (2010), which was kindly provided by the authors. In the case of Bayes A and Bayes B, we used a program described by Hickey and Tier (2009), which is freely available at http://sites.google.com/site/hickeyjohn/alphabayes. For the BRNN, we used the FMB software available at http://www.cs.toronto.edu/∼radford/fbm.software.html. Because the computational time required to evaluate the predictive ability of the BRNN network was great, we used the Condor high throughput computing system at the University of Wisconsin-Madison (http://research.cs.wisc.edu/condor). The RBFNN model was run using Matlab 2010b for Linux. The differences in computing times between the models were great. The computing times for evaluating the prediction ability of the 50 partitions for each trait were as follows, 10 min for RBFNN, 1.5 hr for RKHS, 3 hr for BRR, 3.5 hr for BL, 4.5 hr for Bayes B, 5.5 hr for Bayes A, and 30 days for BRNN. In the case of RKHS, BRR, BL, Bayes A, and Bayes B, inferences were based on 35,000 MCMC samples, and on 10,000 samples for BRNN. The estimated computing times were obtained using, as reference, a single Intel Xeon CPU 5330 2.4 GHz and 8 Gb of RAM memory. Significant reduction in computing time was achieved by parallelizing the tasks.

## RESULTS

Data from replicated experiments in 2010 were used to calculate the broad-sense heritability for each trait in each environment (Table 1). Broad-sense heritability across locations for 2010 data were 0.67 for GY and 0.92 for DTH. These high estimates can be explained, at least in part, by the strict environmental control of trials conducted at CIMMYT’s experiment station at Ciudad Obregon. The heritability of the two traits for 2009 was not estimated because the only available phenotypic data were adjusted means for each environment.

### Predictive assessment of the models

The predictive ability of the different models for GY and DTH varied among the 12 environments. The model deemed best using correlations (Table 2) tended to be the one with the smallest average PMSE (Table 3). The three non-parametric models had higher predictive correlations and smaller PMSE than the linear models for both GY and DTH. Within the linear models, the results are mixed, and all models gave similar predictions. Within the non-parametric models, RBFNN and RKHS always gave higher correlations between predicted values and realized phenotypes, and a smaller average PMSE than the BRNN. The mean of the correlations and the associated standard errors can be used to test for statistically significant improvements in the predictability of the non-linear models *vs.* the linear models. The *t*-test (with α=0.05) showed that RKHS gave significant improvements in prediction in 13/19 cases (Table 3) compared with the BL, whereas RBFNN was significantly better than the BL in 10/19 cases. Similar results were obtained when comparing RKHS and RBFNN with Bayes A and Bayes B.

**Table 2:** Average correlation (SE in parentheses) between observed and predicted values for grain yield (GY) and days to heading (DTH) in 12 environments for seven models

Trait	Environment	BL	BRR	Bayes A	Bayes B	RKHS	RBFNN	BRNN
	1	0.59 (0.11)	0.59 (0.11)	0.59 (0.11)	0.56 (0.11)	0.66 (0.09)	0.66 (0.10)	0.64 (0.11)
	2	0.58 (0.14)	0.57 (0.14)	0.61 (0.12)	0.57 (0.13)	0.63 (0.13)	0.61 (0.13)	0.62 (0.13)
	3	0.60 (0.13)	0.60 (0.12)	0.62 (0.11)	0.60 (0.12)	0.68 (0.10)	0.69 (0.10)	0.67 (0.11)
	4	0.02 (0.18)	0.07 (0.17)	0.06 (0.17)	0.06 (0.17)	0.12 (0.18)	0.16 (0.18)	0.02 (0.19)
DTH	5	0.65 (0.09)	0.64 (0.10)	0.66 (0.09)	0.66 (0.09)	0.69 (0.08)	0.68 (0.08)	0.68 (0.08)
	8	0.36 (0.15)	0.37 (0.15)	0.36 (0.15)	0.35 (0.14)	0.46 (0.13)	0.46 (0.14)	0.39 (0.15)
	9	0.59 (0.12)	0.59 (0.11)	0.53 (0.12)	0.52 (0.11)	0.62 (0.11)	0.63 (0.11)	0.61 (0.12)
	10	0.54 (0.14)	0.52 (0.14)	0.56 (0.13)	0.54 (0.14)	0.61 (0.13)	0.62 (0.12)	0.57 (0.13)
	11	0.52 (0.15)	0.52 (0.16)	0.53 (0.13)	0.51 (0.13)	0.58 (0.14)	0.59 (0.13)	0.55 (0.14)
	12	0.45 (0.19)	0.42 (0.18)	0.45 (0.18)	0.45 (0.18)	0.47 (0.18)	0.39 (0.19)	0.35 (0.19)
	Average	0.59 (0.12)	0.58 (0.12)	0.60 (0.12)	0.57 (0.12)	0.65 (0.10)	0.48 (0.14)	0.48 (0.14)
								
	1	0.48 (0.13)	0.43 (0.14)	0.48 (0.13)	0.46 (0.13)	0.51 (0.12)	0.51 (0.12)	0.50 (0.13)
	2	0.48 (0.14)	0.41 (0.17)	0.48 (0.14)	0.48 (0.14)	0.50 (0.14)	0.43 (0.16)	0.43 (0.16)
	3	0.20 (0.21)	0.29 (0.22)	0.20 (0.22)	0.18 (0.22)	0.37 (0.20)	0.42 (0.21)	0.32 (0.24)
GY	4	0.45 (0.15)	0.46 (0.13)	0.43 (0.15)	0.42 (0.15)	0.53 (0.12)	0.55 (0.11)	0.49 (0.14)
	5	0.59 (0.14)	0.56 (0.16)	0.75 (0.11)	0.74 (0.12)	0.64 (0.13)	0.66 (0.13)	0.63 (0.13)
	6	0.70 (0.10)	0.67 (0.11)	0.73 (0.08)	0.71 (0.08)	0.73 (0.08)	0.71 (0.08)	0.69 (0.10)
	7	0.46 (0.14)	0.50 (0.14)	0.42 (0.14)	0.40 (0.15)	0.53 (0.13)	0.54 (0.14)	0.50 (0.14)
	Average	0.62 (0.10)	0.57 (0.14)	0.69 (0.10)	0.70 (0.09)	0.67 (0.09)	0.56 (0.12)	0.65 (0.10)

Fitted models were Bayesian LASSO (BL), RR-BLUP (BRR), Bayes A, Bayes B, reproducing kernel Hilbert spaces regression (RKHS), radial basis function neural networks (RBFNN) and Bayesian regularized neural networks (BRNN) across 50 random partitions of the data with 90% in the training set and 10% in the validation set. The models with highest correlations are underlined.

**Table 3:** Predictive mean- squared error (PMSE) between observed and predicted values for grain yield (GY) and days to heading (DTH) in 12 environments for seven models

Trait	Environment	BL	BRR	Bayes A	Bayes B	RKHS	RBFNN	BRNN
	1	13.02	13.18	12.72	13.23	11.02	10.85	11.52
	2	11.89	12.37	10.65	11.28	10.19	10.72	10.44
	3	8.18	8.44	7.31	7.59	6.29	6.25	6.63
	4	21.59	22.27	21.79	21.67	21.14	22.64	21.49
DTH	5	8.86	9.23	8.48	8.37	7.95	8.02	8.21
	8	14.72	15.22	14.54	14.58	13.12	13.19	14.81
	9	21.38	21.44	23.71	23.93	20.50	19.84	20.62
	10	7.72	8.51	7.27	7.57	6.66	6.51	7.36
	11	6.83	7.12	6.59	6.74	6.03	5.96	6.51
	12	13.60	14.42	13.56	13.46	13.25	14.86	15.75
	Average	6.09	6.47	5.99	6.28	5.31	9.12	9.25
								
	1	0.07	0.09	0.07	0.07	0.07	0.07	0.07
	2	0.06	0.08	0.06	0.06	0.06	0.07	0.07
	3	0.06	0.07	0.06	0.06	0.05	0.05	0.05
GY	4	0.22	0.24	0.23	0.23	0.20	0.19	0.21
	5	0.39	0.44	0.26	0.27	0.35	0.33	0.36
	6	0.13	0.15	0.12	0.13	0.12	0.13	0.13
	7	0.40	0.41	0.43	0.44	0.38	0.37	0.39
	Average	0.06	0.07	0.05	0.05	0.05	0.07	0.06

Fitted models were Bayesian LASSO (BL), RR-BLUP (BRR), Bayes A, Bayes B, reproducing kernel Hilbert space regression (RKHS), radial basis function neural networks (RBFNN) and Bayesian regularized neural networks (BRNN) across 50 random partitions of the data with 90% in the training set and 10% in the validation set. The models with lowest PMSE are underlined.

**Figure 3:**
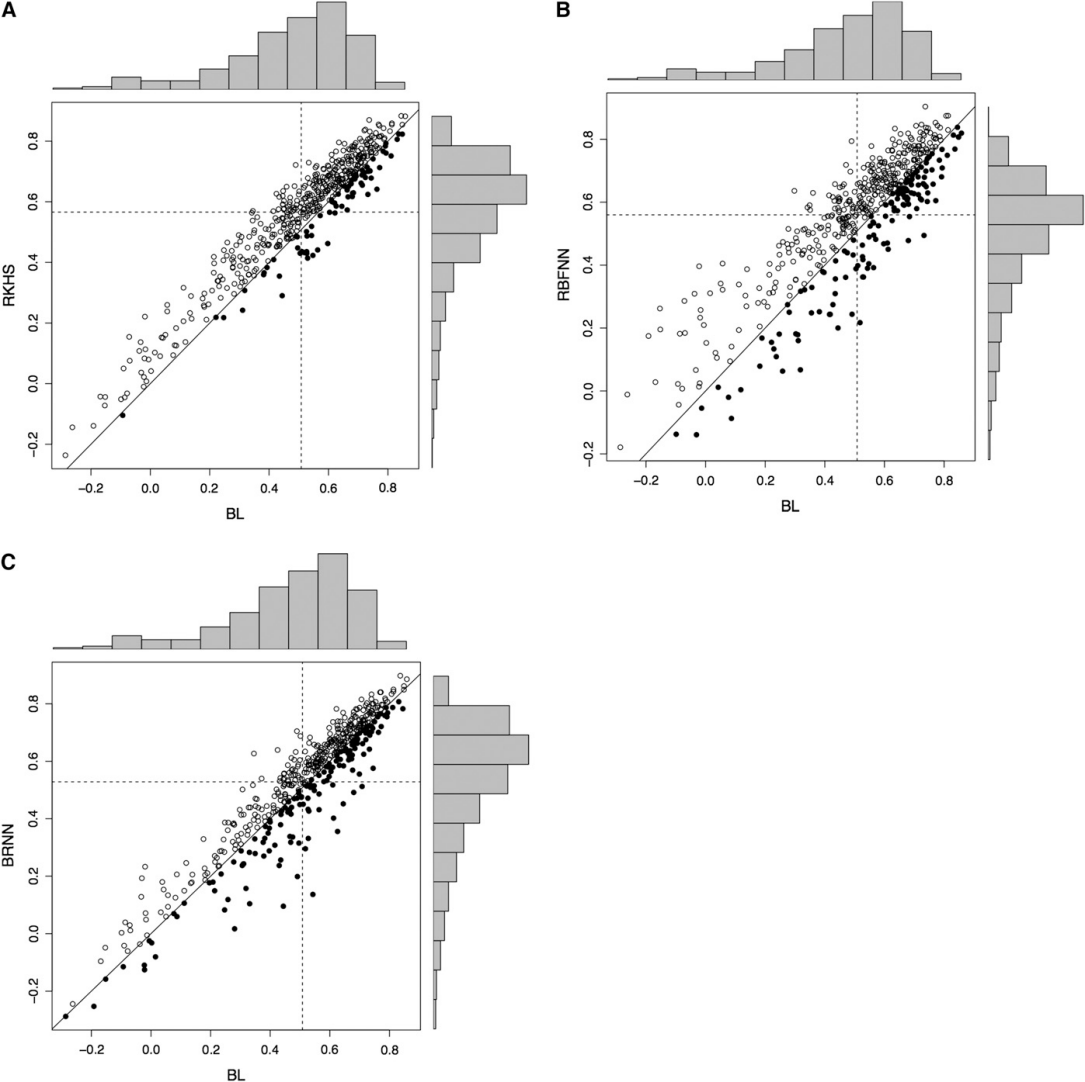
Plots of the predictive correlation for each of 50 cross-validation partitions and 10 environments for days to heading (DTH) in different combinations of models. (A) When the best non-parametric model is RKHS, this is represented by an open circle; when the best linear model is BL, this is represented by a filled circle. (B) When the best non-parametric model is RBFNN, this is represented by an open circle; when the best linear model is BL, this is represented by a filled circle. (C) When the best non-parametric model is BRNN, this is represented by an open circle; when the best linear model is BL, this is represented by a filled circle. The histograms depict the distribution of the correlations in the testing set obtained from the 50 partitions for different models. The horizontal (vertical) dashed line represents the average of the correlations for the testing set in the 50 partitions for the model shown on the Y (X) axis. The solid line represents Y = X; *i.e.* both models have the same prediction ability.

**Figure 4:**
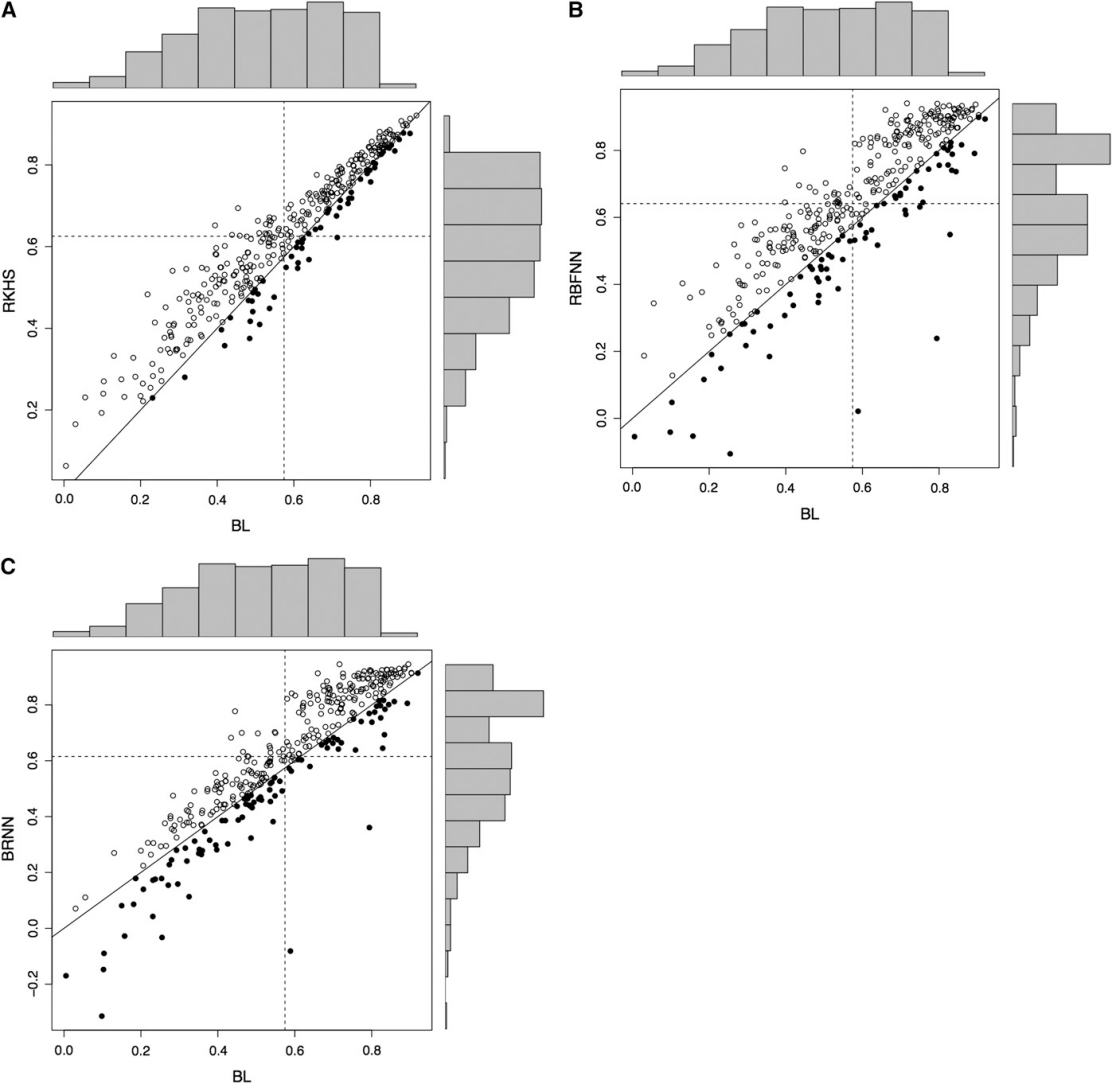
Plot of the correlation for each of 50 cross-validation partitions and seven environments for grain yield (GY) in different combinations of models. (A) When the best model is RKHS, this is represented by an open circle; when the best model is BL, this is represented by a filled circle. (B) When best model is RBFNN, this is represented by an open circle; when the best model is BL, this is represented by a filled circle. (C) When the best model is BRNN, this is represented by an open circle; when the best model is BL, this is represented by a filled circle. The histograms depict the distribution of the correlations in the testing set obtained from the 50 partitions for different models. The horizontal (vertical) dashed line represents the average of the correlations for the testing set in the 50 partitions for the model shown on the Y (X) axis. The solid line represents Y = X; *i.e.* both models have the same prediction ability.

Correlations between observed and predicted values for DTH were lowest overall in environments 4 and 8, in Cd. Obregon, 2009, and in Toluca, 2009. Average PMSE was in agreement with the findings based on correlations. Although accuracies in environment 4 were much lower than in other environments, the higher accuracy of the non-parametric models (RKHS, RBFNN, and BRNN) over that of the linear models (BL, BRR, Bayes A, and Bayes B) was consistent with what was observed in the other environments. Figures 3 and 4 give scatter plots of the correlations obtained with the three non-parametric models *vs.* the BL for DTH and GY, respectively; each circle represents the estimated correlations for each of the two models included in the plot. In Figure 3, A–C, DTH had a total of 500 points (10 environments and 50 random training-testing partitions). In Figure 4, A–C, GY had a total of 350 points (7 environments and 50 random partitions in each environment). A point above the 45-degree line represents an analysis where the method whose predictive correlation is given on the vertical axis (RKHS, RBFNN, BRNN) outperformed the one whose correlation is given on the horizontal axis (BL). Both figures show that although there is a great deal of variability due to partition, for both DTH and GY, the overall superiority of RKHS and RBFNN over the linear model BL is clear. For both traits, BL had slightly better prediction accuracy than the BRNN in terms of the number of individual correlation points. It is interesting to note that some cross-validation partitions picked subsets of training data that had negative, zero, or very low correlations with the observed values in the validation set. These results indicate that lines in the training set are not necessarily related to those in the validation set.

## DISCUSSION AND CONCLUSIONS

Understanding the impact of epistasis on quantitative traits remains a major challenge. In wheat, several studies have reported significant epistasis for grain yield and heading or flowering time (Goldringer *et al.* 1997). Detailed analyses have shown that vernalization, day-length sensitivity, and earliness *per se* genes are mainly responsible for regulating heading time. The vernalization requirement relates to the sensitivity of the plant to cold temperatures, which causes it to accelerate spike primordial formation. Transgenic and mutant analyses, for example, have suggested a pathway involving epistatic interactions that combines environment-induced suppression and upregulation of several genes, leading to final floral transition (Shimada *et al.* 2009).

There is evidence that the aggregation of multiple gene × gene interactions (epistasis) with small effects into small epistatic networks is important for explaining the heritability of complex traits in genome-wide association studies (McKinney and Pajewski 2012). Epistatic networks and gene × gene interactions can also be exploited for GS via suitable statistical-genetic models that incorporate network complexities. Evidence from this study, as well as from other research involving other plant and animal species, suggests that models that are non-linear in input variables (*e.g.* SNPs) predict outcomes in testing sets better than standard linear regression models for genome-enabled prediction. However, it should be pointed out that better predictive ability can have several causes, one of them the ability of some non-linear models to capture epistatic effects. Furthermore, the random cross-validation scheme used in this study was not designed to specifically assess epistasis but rather to compare the models’ predictive ability.

It is interesting to compare results from different predictive machineries when applied to either maize or wheat. Differences in the prediction accuracy of non-parametric and linear models (at least for the data sets included in this and other studies) seem to be more pronounced in wheat than in maize. Although differences depend, among other factors, on the trait-environment combination and the number of markers, it is clear from González-Camacho *et al.* (2012) that for flowering traits (highly additive) and traits such as grain yield (additive and epistatic) in maize, the BL model performed very similarly to the RKHS and RBFNN. On the other hand, in the present study, which involves wheat, the RKHS, RBFNN, and BRNN models clearly had a markedly better predictive accuracy than BL, BRR, Bayes A, or Bayes B. This may be due to the fact that, in wheat, additive × additive epistasis plays an important role in grain yield, as found by Crossa *et al.* (2006) and Burgueño *et al.* (2007, 2011) when assessing additive, additive × additive, additive × environment, and additive × additive × environment interactions using a pedigree-based model with the relationship matrix **A**.

As pointed out first by Gianola *et al.* (2006) and subsequently by Long *et al.* (2010), non-parametric models do not impose strong assumptions on the phenotype-genotype relationship, and they have the potential of capturing interactions among loci. Our results with real wheat data sets agreed with previous findings in animal and plant breeding and with simulated experiments, in that a non-parametric treatment of markers may account for epistatic effects that are not captured by linear additive regression models. Using extensive maize data sets, González-Camacho *et al.* (2012) found that RBFNN and RKHS had some similarities and seemed to be useful for predicting quantitative traits with different complex underlying gene action under varying types of interaction in different environmental conditions. These authors suggested that it is possible to make further improvements in the accuracy of the RKHS and RBFNN models by introducing differential weights in SNPs, as shown by Long *et al.* (2010) for RBFs.

The training population used here was not developed specifically for this study; it was made up of a set of elite lines from the CIMMYT rain-fed spring wheat breeding program. Our results show that it is possible to achieve good predictions of line performance by combining phenotypic and genotypic data generated on elite lines. As genotyping costs decrease, breeding programs could make use of genome-enabled prediction models to predict the values of new breeding lines generated from crosses between elite lines in the training set before they reach the yield testing stage. Lines with the highest estimated breeding values could be intercrossed before being phenotyped. Such a “rapid cycling” scheme would accelerate the fixation rate of favorable alleles in elite materials and should increase the genetic gain per unit of time, as described by Heffner *et al.* (2009).

It is important to point out that proof-of-concept experiments are required before genome-enabled selection can be implemented successfully in plant breeding programs. It is necessary to test genomic predictions on breeding materials derived from crosses between lines of the training population. If predictions are reliable enough, an experiment using the same set of parental materials could be carried out to compare the field performance of lines coming from a genomic-assisted recurrent selection program scheme *vs.* lines coming from a conventional breeding scheme. The accuracies reported in this study represent prediction of wheat lines using a training set comprising lines with some degree of relatedness to lines in the validation set. When the validation and the training sets are not genetically related (unrelated families) or represent populations with different genetic structures and different linkage disequilibrium patterns, then negligible accuracies are to be expected. It seems that successful application of genomic selection in plant breeding requires some genetic relatedness between individuals in the training and validation sets, and that linkage disequilibrium information *per se* does not suffice (*e.g.* Makowsky *et al.* 2011).

## Supplementary Material

Supporting Information
