# Supplementary material for: Comparison Between Linear and Non-parametric Regression Models for Genome-Enabled Prediction in Wheat
Source: G3 (Bethesda). 2012 Dec 1;2(12):1595–605. doi: 10.1534/g3.112.003665 (PMC3516481; doi:10.1534/g3.112.003665)
Supplement: Supporting Information [file supp_2_12_1595__index.html]

Supporting Information 

# Comparison Between Linear and Non-parametric Regression Models for Genome-Enabled Prediction in Wheat

## Supporting Information for Perez-Rodriguez *et al.*, 2012

**Files in this Data Supplement:**

- File S1 - Supporting Data (.zip, 3.6 MB)
